# The prevalence of foot-and-mouth disease in Asia

**DOI:** 10.3389/fvets.2023.1201578

**Published:** 2023-06-30

**Authors:** Munazza Aslam, Khalid A. Alkheraije

**Affiliations:** ^1^Department of Pathology, Faculty of Veterinary Science, University of Agriculture, Faisalabad, Pakistan; ^2^Department of Veterinary Medicine, College of Agriculture and Veterinary Medicine, Qassim University, Buraidah, Saudi Arabia

**Keywords:** FMD, virus, prevalence, geographical distribution, pathophysiology, prevention

## Abstract

Foot-and-mouth disease (FMD) is listed among the highly contagious diseases in animals and is endemic throughout the Asian continent. The disease is caused by the Foot-and-mouth disease virus (FMDV) and affects a wide variety of domesticated animals as well as wild ungulates. Clinically, the disease is described as a vesicular lesion on the tongue, muzzle, lips, gum, dental pad, interdigital cleft, coronary band, and heel of the foot. Sometimes these lesions give rise to lameness. Mastitis is also caused due to teat lesions. A biochemical test reveals that during FMD infection, there are elevated levels of interleukin-1 (IL-1), tumor necrosis factor-alpha, interferon-gamma (IFN-γ), interleukin-6, serum amyloid A protein, lactoferrin, mannose-binding lectin, and monocytes chemo-attractant protein-1 in the serum of infected animals. There is no specific treatment for FMD although some antivirals are given as prophylaxis and antibiotics are given to prevent secondary bacterial infection. This review presents comprehensive data on the prevalence of FMD and serotypes of FMDV that are attributable to the cause of FMD from a regional point of view. It also explains the worldwide dynamics of the seven serotypes of FMD and tries to identify epidemiological clusters of FMD in various geographical areas. Furthermore, the pathology associated with the foot and mouth disease virus along with the pathophysiology is discussed. The continent-wide prevalence and diversity patterns of FMD suggest that there is a need for stringent policies and legislation implementation regarding research and development aimed at manufacturing strain-specific vaccination, infection prevention, and control of the disease.

## 1. Introduction

Foot-and-mouth disease (FMD) is a highly contagious disease caused by the foot-and-mouth disease virus (FMDV). This virus belongs to the genus *Aphthovirus* of the *Picornaviridae* family (1). Cloven-hoofed animals such as buffaloes, cattle, goats, sheep, and pigs are affected by FMD (2). The virus is composed of a single copy of the RNA genome (3). This RNA is positive sense having a length of 8,400 nucleotides (2, 4). This RNA genome is surrounded by four separate structural proteins named VP1, VP2, VP3, and VP4. These four proteins form a capsid around the genome. Antigenic properties are attributed to the virus because of VP1, VP2, and VP3 as they are externally present while VP4 is present internally in the capsid (4). FMDV has seven serotypes (O, A, C, Asia 1, SAT 1, SAT 2, and SAT 3) reported from different regions of the world (5). There are certain characteristics that make the animals prone to disease such as their incubation period of 1–2 weeks (6), capability to withstand harsh environments, low infectious dose (7), and rapid viral replication in the host. Nasal secretions have a high viral titer which is responsible for disease transmission to susceptible animals (8). Certain factors affect the disease-causing ability of FMDV such as abrasions by which the virus enters the body, the release of virus titer, and the duration the virus stays in the body. Besides, aerosol and mechanical routes also play a vital part in the dissemination of the disease (9). The situation is aggravated due to the uncontrolled movement of animals across different states or countries (8). Pathogenesis is attributed to integrin and heparan sulfate receptors which allow FMDV to enter the host cell, and after replication by the host cell, lysis virus is released into neighboring cells.

Ungulates and wild animals are affected by FMD which results in the formation of vesicular lesions on the hoof and mouth. It is a highly contagious and transboundary disease. It is endemic in most Asian countries, imposing a serious threat to the economy of these countries. Indonesia and the Philippines are FMD-free countries in Asia (10). It results in high morbidity and mortality in adults and juveniles, respectively (11). Clinical signs observed in infected animals are anorexia, fever, and excessive salivation, as well as the formation of vesicular blisters over the nose, muzzle, tongue, teats, feet, snout, and other glabrous skin parts, which ultimately result in lameness (11). The risk of mastitis increases in those animals that develop lesions on their teats. In the susceptible population, the disease rate is 100%. Young suckling calves have a 100% fatality rate, and death due to myocarditis may increase up to 50% (12). Animals infected by FMD may become carriers of disease after their recovery and are a potential threat to healthy animals because of the persistence of the virus in the animal. This persistence is also affected by the physical state of the host (13, 14). Wildlife having cloven feet are also infected by FMDV (15). Proper vaccination and quarantine of infected animals are done to prevent the disease from spreading (16). In FMD-free countries, they have culled several animals to get to a disease-free state (17).

Vast geographic distribution, diversity among viral serotypes, and high affinity of the virus to cause the disease are the factors that make the virus economically important (16). Office International des Epizooties (OIE) has listed FMD among notifiable diseases because of its cross-border dispersal, high infectivity, and transmission power (18). The trafficking ban has made FMD a hidden dairy and meat industry enemy. Milk and meat production is reduced ultimately, leading to disease and resulting in economic loss (19). In February 2020, the Ministry of Planning and Special Initiatives demonstrated that the annual loss has exceeded 629 million USD due to FMD in dairy animals in Pakistan (20). Outbreaks are also reported from different countries, i.e., Saudi Arabia, Korea, Libya, India, and Iran. The persistence of FMDV in Asian countries can paralyze the livestock sector by negatively impacting the economy and agriculture (5). Therefore, this review is done to determine the prevalence of FMDV so steps can be taken to control the disease and the devastating effects of the disease on the economy of Asian countries.

## 2. Characteristics of FMDV

**Figure 1:**
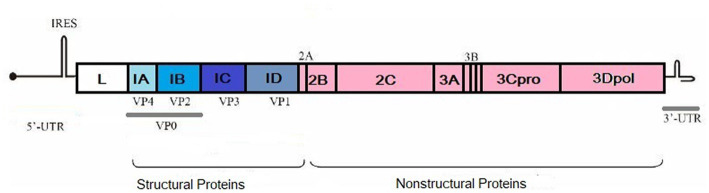
Structural and non-structural proteins of FMDV. Created by biorender.com.

FMDV has seven distinct serotypes, and they do not induce any cross-protection against each other. Serotypes are named A, O, C, Asia 1, South African Territories 1 (SAT 1), SAT 2, and SAT 3 (21). Since 2004, serotype C has not been detected and is thought to be extinct. In 2004, it was reported from Kenya and Brazil (22). All the serotypes are further classified into different lineages that are distinct from each other and do not induce efficient cross-protection from the same serotypes of other viruses (23). Serotype identification is done based on the nucleotide sequence of the VP1 protein. Differences among the coding sequences in VP1 give rise to different sublineages, lineages, and topotypes (24). Serotype O has a history of worldwide outbreaks. The African region has all the serotypes except Asia 1. Serotypes A, O, and Asia 1 are reported to cause outbreaks in Asia. Middle East and African countries harbor SAT 1 and SAT 2 (22). The FMDV genome consists of 8,500 nucleotides, and it contains an open reading frame (ORF). At both ends of ORF, 5′ and 3′-untranslated regions (UTR) are present. A polyprotein of 2,300 amino acids is encoded by ORF which is further processed by viral proteases. This processing leads to the formation of mature viral proteins and precursors. Four structural and 10 nonstructural proteins are made during this process (Figure 1, produced by biorender).

## 3. The distribution pattern of FMDV in Asian countries

**Figure 2:**
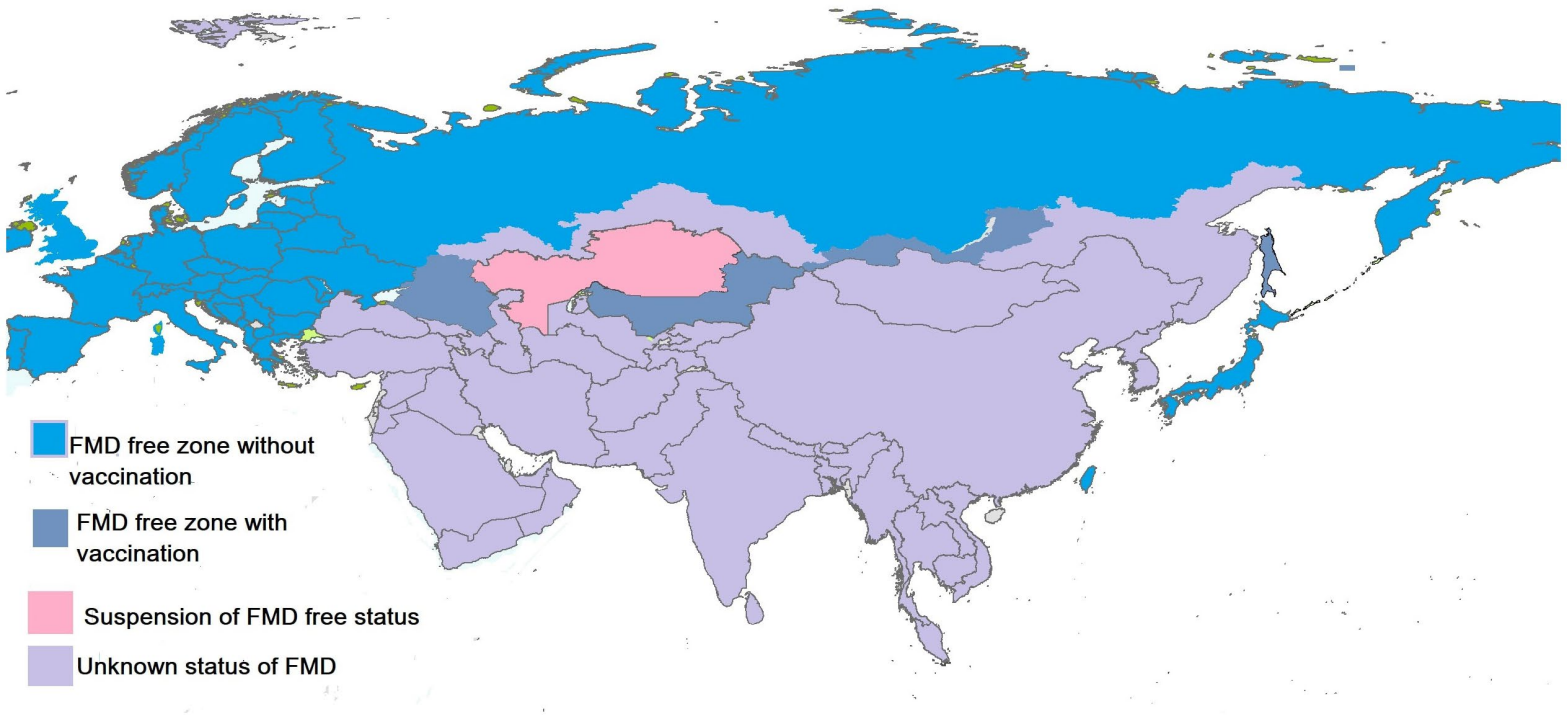
Status of FMDV in Asian countries. Created by biorender.com.

FMDV has prevailed in different regions of Asia and is endemic in many countries. These countries have a history of a wide range of outbreaks of FMD. The status of FMD in the Asian region illustrated by OIE in September 2022 is shown in Figure 2. The distribution pattern of the disease is described in the respective sections along with the factors that aid in the persistence of the disease in a particular region.

### 3.1. Pakistan

FMD has been a headache for livestock holders for many decades. Many studies are conducted to reveal the hidden mechanism to overcome the virus. The prevalence and molecular characterization of the virus are studied by many scientists for vaccine production, but still, it is endemic in the region. A total of 1,478 small ruminants were tested for seroprevalence of FMD. Seroprevalence was reported to be 22.8% (25). In another study conducted on sheep, mortality reported with FMD was 48.88% and the serotype reported was Asia 1 (26). In Khyber Pakhtunkhwa, serum samples from 2,511 animals were collected and 9.83% were positive for FMD (27). FMD was reported in captive yaks that were kept at a wildlife park. Prevalence was reported to be 75% and were infected by serotype A (28). The Punjab region is notorious for FMD as it is an active region in terms of animal trade, and its environment favors viral propagation (29). From Islamabad, in a study conducted to check the prevalence of FMDV among buffaloes, 77.7% of 300 buffaloes were positive for anti-FMD antibodies. Persistent infection was present in eight buffaloes and serotype Asia 1 was most prevalent followed by the A and O serotypes (30). Molecular characterization of FMD serotypes was done and they reported the prevalence of serotype A followed by Asia 1 (31). Recently, an outbreak was reported in different districts of Punjab. From the Multan district, 26 outbreaks were reported, with serotype O being the most prevalent followed by Asia 1 and A with a prevalence of 45.83%, 29.17%, and 13.89%, respectively. There were cases of mixed infection of serotype Asia 1 and O (1.39%) and serotypes O and A (9.72%) (30). Still, FMD is endemic in the region and a menace to the economy of the country.

### 3.2. India

FMD is an endemic infection in India affecting livestock and the serotype circulating in the region is O, A, and Asia 1. Geographically, the prevalence of FMD was reported to be 31.5%, 43%, 11.6%, 5%, 4.4%, and 4% in the Southern, Eastern, North-eastern, Central, Western, and Northern regions, respectively (32). In a research study of the Odisha region, antibodies against structural proteins (SP) and non-structural proteins (NSP) were tested and the result revealed that antibodies against NSP were higher in goats (38.33%) followed by cattle (33.33%) and sheep (3.93%). Antibodies against SP were 68.62% in cattle, 38.87% in goats, and 17.32% in sheep (33). To check the prevalence of FMD, 41,009 samples from 39 studies were tested and it was reported to be 21% in the northeastern region of India (34). O/ME-SA/Ind2001e, a sub-lineage of serotype O, was most prevalent from 2014 to 2018; its evolutionary rate was also faster than sub-lineage O/ME-SA/Ind2001d. A new sublineage among O serotypes was also identified and named O/ME-SA/2018. Its sustenance and prevalence need to be studied (35). A 3-year study was done to check the effect of vaccination of FMD in Karnataka state. It revealed a decrease in the seroprevalence rate of disease from 58 to 21%. Immunity was recorded in animals against O, A, and Asia 1 serotypes, and it was boosted from 4.5 to 59% in animals (36). In the past decade, most outbreaks occurred in 2013 and 2018 while the least number of cases were reported in 2022. Month-scale analysis for the prevalence of FMD revealed that the highest incidence of the disease was between October and March (37). The Effect of mass vaccination was determined to check the seroprevalence of the disease; more than 1 million animals were sampled and the result revealed a 50% reduction in the incidence of FMD (38). Climatic conditions such as monsoons and the transboundary movement of animals are a hindrance in the eradication of the disease from its origin. Furthermore, the intermixing of different serotypes has also been a headache in the preparation of vaccines as they lose their seron-specificity when a novel virus serotype is formed. Disturbance in the cooled chain and inadequate storage leads to the deterioration of the virus resulting in vaccine failure. FMD control program is working in India to eradicate the disease by 2030 as they have eradicated the polio virus and rinderpest with the same method (39).

### 3.3. Afghanistan

Over the past few decades, conflicts have been ongoing in Afghanistan that have resulted in the massive destruction of the infrastructure of the country. Illegal transboundary movement of livestock between the Pak and Afghan border has boosted the spread of FMD. Serotypes O, A, and Asia 1 are endemic in the region, O being the most prevalent serotype. The study has shown that the O serotype has different sub-lineages that are circulating in the country namely Pak98, Iran2001, and PanAsia (40). Outbreaks of FMD from 1995 to 2016 were studied to check the distribution of the disease in the country. Between 1995 and 2008, the total number of outbreaks reported was 4,171. A total of 7,558 samples were collected from 34 provinces, and 54.1% were positive for anti-FMDV antibodies. Prevalence varied among 2009, 2011, and 2013 to 2015 significantly. In 2016, clinically suspected cases of FMD were reported (41). FMDV prevalence was further confirmed by researchers who conducted a study on clinically suspected cases from the Civil Veterinary Hospital situated in the Nangarhar province (42). In the Baghlan province where the research study was conducted, 376 cattle from nearly 200 herds were sampled from 53 villages of Doshi, Puli Khumri, and Khinian districts, and seroprevalence was estimated to be 20% (43). FMD is difficult to eradicate from these regions because of various factors like socio-economic disruption, droughts most markedly draught from 1998 to 2001, and the lack of a stable government in past decades (44). The disease surveillance system adapted is of a passive nature which does not help in proper disease investigation. The lack of an Epidemiological Unit to keep up with the livestock information is considered a major constraint in the eradication of FMD in Afghanistan (45).

### 3.4. Bangladesh

FMD is an endemic disease in Bangladesh affecting a wide variety of cloven-footed animals. In a study, the prevalence of FMD in the Rajshahi region was reported to be 25.07%. This study also revealed that animals that were kept in rural household farming systems (26.03%) were at a higher risk of FMD than in intensive farming systems (23.44%) (46). In another study, 21 cases of FMD were reported at the teaching hospital of the Jhenaidah district of Bangladesh. From Meghna Upazila, the prevalence of FMD was 24.51% (47). In 2014, 153,421 cases of FMD were reported, and in 2015, the number of cases was reduced to 102,767. In 2016, a rise in the number of cases occurred and 140,270 infected animals were reported. In 2017, this number increased to 152,359 (48). In the Haor areas of Bangladesh, the prevalence of FMD was calculated to be 24.71% among cattle (49). In regions with climatic conditions such as heavy rainfall, they are more exposed to FMD and are the high-risk areas of the country. For resource-limited settings, vaccination should be done primarily in hotspot areas having a high prevalence rate of the disease. During surveillance, the eastern regions of Bangladesh should be specially targeted in the post-monsoon season (48).

### 3.5. China

In 1958, FMDV was first reported from the Xinjiang Uyghur region of China; serotypes O and A were prevalent in these regions while Asia 1 was reported from the Yunnan province of China (50). In 2005, serotype Asia 1 was again detected in cattle in the Wuxi, Jiangsu province. Between 2005 and 2009, FMD expanded to mainland China and affected 17 provinces (51). The Asia 1 serotype was eradicated in 2009, and since 2010, only serotypes O and A have been observed in northwestern and southeastern China (52). Since 2010, 140 outbreaks have been reported in China. In 2020, about 70 cattle infected with FMDV were reported from Heshuo County, and six FMDV-infected cattle were reported from Xinjiang Uyghur Autonomous Region. The highest number of outbreaks is seen in the region of Tibet and Xinjiang. This may be due to the presence of susceptible species and a high population density (52). FMD outbreaks (109 in number) were assessed, and studies were performed to check the prevalent serotype and its associated risk factors. The serotype reported from mainland China was clusters of A and O (53). Rural farming at a smaller scale and open grazing are the risk factors that increase the chances of FMD in the region. Furthermore, climate, breeding factors, livestock density, and transportation to different provinces play a key role in the spread of the disease (51, 54).

### 3.6. Mongolia

Since 1973, FMD was not prevalent in Mongolia till 2000; cases of FMD were reported between April 2000 and July 2002. A total of 44 outbreaks of FMD were reported that infected camels, goats, sheep, and cattle. A study was conducted to check the antibody status, and 2% of four livestock specie were positive for antibodies against nonstructural proteins, whereas for structural proteins it was 30.3%. In 2008, a significant decline in the antibody titer was found, indicating a decline in antibodies against FMDV (55). FMD is not an endemic disease; it is occasionally seen in the Eastern region and spreads to other parts of the country that are FMD-free (55, 56). Serotypes O and A are prevalent in Mongolia and the most affected specie is cattle followed by sheep and goats (55, 57). Disease evidence is also reported in Bactrian camels and wildlife (58–60). A rise in FMD cases was reported in 2017–2018, with multiple lineages of serotype O and a single lineage of serotype A (61). In this outbreak, virus isolation was done from field cases of Bactrian camels for the first time (62). A workshop was conducted for the eradication of FMD in the region. The workshop participants came up with a list of almost 80 potential ideas to enhance risk management after completing the risk calculation for all pathways. However, a remarkably high level of agreement was attained with the ranking by identifying the four most crucial recommendations in each part. The need to raise FMD awareness among herders, the general public, and veterinarians was seen to be of the utmost importance. Strengthening the system for regulating cattle movement was also regarded as essential, with proposals addressing various facets of border control and the issuance of health and origin certifications for internal movements. It was advised to stick with a risk-based immunization strategy for prevention in risk areas by concentrating on locations near the main transportation routes from the border to provincial centers (63).

### 3.7. Kazakhstan

In 2012 sheep, goats, and cattle were tested for the presence of anti-FMDV antibodies. Among 76,851 samples, 8% tested positive. Organs from clinically ill patients were collected and serotyping of the virus was done. It revealed serotype O among all regions except Zhambyl where serotype A22 was present (64). From the era of 1955 to 2013, a total of 5,260 outbreaks were reported, having both serotypes A and O among ungulates. The study concluded spatiotemporal clusters only before 1970; after that era, ring vaccination was employed which eventually prevented FMD epidemics. Disease numbers reduced significantly after ring vaccination and culling the carrier and infected animals (65). In 2017, OIE declared Kazakhstan an FMD-free country, but later in 2022, an outbreak of FMD was reported from the Shetskiy district of Qaraghandy. The implementation of regulated transboundary movement is necessary to control the disease and to gain the FMD-free status of the country (10).

### 3.8. Russia

Russia is a country with a wide geographical area and a big agricultural country. FMD is endemic in the region and has resulted in many outbreaks during the era. They have become a hindrance in the trade of animal products and animals. In a study, it was concluded that the exported cattle of Russian origin have the risk of FMDV because of the presence of the infection in the region (66). In a study conducted on the prevalence of FMD in the last decade, it was reported that in Primosky Krai, the highest morbidity was seen in pigs, while in Amur Oblast, it was in cattle. The epidemic rate was at the highest in Zabaykalsky Krais and Primorsky. A total of 68 outbreaks were reported from 2010 to 2019, and the highest contagiousness was recorded in Primorsky Krai when the FMD outbreak hit many large pig farms in 2014 and 2019 (67). In the past two decades, 97 outbreaks of FMD have been reported. O, A, and Asia 1 serotypes were detected among pigs, cattle, and small ruminants. The Russian-Chinese border is notorious for most of these outbreaks. O and A serotypes accounted for 79% of the FMD outbreaks, with time in 2005 and 2013–2019 clusters of time–space also observed. Mixed serotype clusters lasted for more periods (552 days) than infection by a single serotype (68). Proper legislation and epidemiological surveillance are required to eradicate the disease. Controlled animal transboundary movement along with the controlled movement of visitors on the farm is required to block the spread of disease in other farms of the nearby region.

### 3.9. Egypt

FMD is endemic in Egypt with three distinct serotypes O, A, and SAT 2 (69, 70). It was reported for the first time in 1950 by the World Reference Laboratory for Foot and Mouth Disease. Only two serotypes were prevalent at that time, SAT 2 and O (71, 72). Serotype A was reported for the first time in 1952 and it was again detected in 2006 when livestock was imported from Ethiopia. Serotype SAT 2 also vanished after 1950 and was detected in 2012. This strain was closely related to the Sudan 2008 strain (73–76). New variants among serotype O and SAT2 have been reported in the past few years (77, 78). The relationship of parasitic diseases with FMD was also studied, but no significant relation was detected (79). A new sublineage of SAT 2 was identified in 2019 that resulted in the appearance of drastic clinical signs in buffaloes (80) Seroprevalence of FMD in a study was analyzed for 2021 and 2022. In 2021, it was 48.30% and in 2022 it increased to 68.10% (81). One of the major reasons for FMD being endemic in the region is the illegal transboundary movement of animals and livestock imports. Anti-FMDV antibodies were detected in the cattle imported to Egypt from Sudan (82). In illegally imported animals, seropositivity was 50% (83). Risk analysis has been performed by researchers and they claim a significant decrease in the probability of the disease with the help of vaccination (84). The identification of risk factors linked with such endemic diseases, combined with vaccine application, may aid in infection control in Egypt.

### 3.10. Iran

In Iran, 1,381 outbreaks of FMD have been reported between April 2014 and March 2015. Among these 1,381, 314 outbreaks were in small ruminants while 1,067 were in cattle. A, Asia 1, and O were the prevalent serotypes (81). The Khorasan Razavi province was investigated by the Iranian Animal Disease Department for FMD outbreaks and among 127 farms, 46 were positive (85). In 2017, outbreaks of FMD were reported in Qom province. Phylogenetic analysis of the virus was done and Asia 1 was reported to be prevalent along with O and A serotypes (86, 87). A high level of homology between sublineages of Asia 1 was detected in Pakistan, India, Turkey, and Israel (87). Among 42 clinical samples collected from 16 provinces, serotype A was detected using a sandwich Enzyme-Linked Immunosorbent Assay (ELISA) (88). The internalization and replication of FMDV have been reported in dogs, highlighting the risk of feeding FMD-infected animal carcasses to other animal species (89).

### 3.11. Iraq

FMD is endemic in Iraq. Serotypes O, A, SAT, and Asia 1 were reported from the region (90–93). Outbreaks of FMD were reported in small ruminants, cattle, and buffaloes from 15 Iraqi governorates except for Kurdistan. The disease rate increased significantly in 2016 in comparison to 2015 (94). In 2019, from outbreaks in the Nineveh province of Iraq, molecular characterization and prevalence rates were determined, and 46.95% and 40.43% were the prevalence rates of FMD, using ELISA and RT-PCR technology (95). In Mosul city of Iraq, seroprevalence among calves was reported to be 48.64% (96). FMD results in tremendous financial damage to livestock owners on an annual basis as a part of the endemicity discussed previously in the respective section. A review of scientific literature showed a scarcity of publications on the epidemiology of FMD in Iraq (94). Furthermore, there are suitable plans for providing vaccines to farmers, but implementation on the ground is limited. Furthermore, the open market is overrun with uncontrolled vaccines of unknown efficacy, and unrestricted movement of animals between Iraq’s governorates results in the persistence of the disease in the region.

### 3.12. Kuwait

During the time lapse between 2005 and 2020, a total of nine outbreaks of FMD were reported from the Jahra district of Kuwait. A surveillance system was developed for the early detection of the disease. In 2009, more than 2000 susceptible cases of FMD appeared but only 60 were registered in Sulaibiya. Again, an outbreak of FMD occurred in 2011. In 2012, two outbreaks occurred at the Kuwait-Iraq and Kuwait-Saudi border. In 2016, 711 confirmed FMD cases were reported, and 6,101 cases were at risk. Till 2020, outbreaks have been reported from the center, north, and south of Kuwait (97). For disease control, an established early detection system was used as a surveillance and monitoring instrument to determine the state of local animal health, allowing for the rapid identification of disease outbreaks and the monitoring of disease spread patterns (97). The early detection system aids in the visualization of disease outbreaks, allowing for continuous updates on the disease situation and interventions if animal health risks arise (98). The system can also provide useful information for advanced methods that support animal health surveillance. Kuwait was the first country to suggest the establishment of an early warning center among the Gulf Cooperation Council (GCC) countries. In 2012, the “Gulf Early Warning Center for Transboundary Animal Diseases” was created under the supervision of the deputy director general for livestock for the eradication of FMD (97).

### 3.13. Saudi Arabia

FMD being endemic in the Saudi region has been a headache for the economy of the country, and Serotypes O, A, and Asia 1 have been reported from the region. A new lineage of serotype O (O/ME-SA/Ind-2001) has been reported during an outbreak of FMD in Libya and the Saudi Arabia region (99). An outbreak was also reported at sheep farms resulting in the abortion and death of neonates (100). Milk samples were examined for the presence of FMDV RNA. It was detected in 5.7% (42/732) of the milk samples (101). In Hail, seroprevalence of FMD was reported to be 17.5% in non-vaccinated animals (102). The requirement to confirm the effectiveness of present animal health interventions needs to be emphasized. There must also be a uniform FMD immunization plan. Following a primary immunization at 4 months of age, a booster shot at 5 months and herd vaccination every 4 months are advised.

It will be possible to continuously monitor the disease across Saudi Arabia because of established local laboratory facilities to look into the current condition of FMD in the country to ensure that the used vaccination can protect against the FMD viruses circulating in Saudi Arabia. This necessitates a continuous effort to raise animal owners’ and dairy farm managers’ knowledge of the importance of cooperating with the Veterinary Authorities of the Ministry of Agriculture (103).

### 3.14. Oman

From 2011 to 2015, a total of 64 outbreaks of FMD were investigated to check the serotype of FMDV, and it was later revealed by testing that it was serotype SAT2. Serotypes O, A, and Asia 1 were previously reported from the region. FMD being endemic in the Saudi region has been a headache for the economy of the country and Serotypes O, A, and Asia 1 have been reported from the region (104). However, in camels, it was not reported even when kept with other infected livestock species (105). Factors increasing direct and indirect contact between herds and wildlife, such as mixing at shared pastures or watering sites, were frequently reported at the herd level. Mixed herd practices have been adapted widely in Oman, which play a significant part in disease transmission from one animal to another.

### 3.15. United Arab Emirates

Serotypes O, A, and Asia1 have been reported from outbreaks of FMD in the 1980s in the United Arab Emirates (UAE) (106). Serosurvey was done in Arabian Oryx against many viral diseases but FMD was not reported among them (107). In the UAE, the Camel of Bactrian and Dromedary species are significantly different regarding susceptibility to FMDV. Bactrian camels are more prone to FMDV while dromedaries are not susceptible and they do not transmit the infection even when being in contact with susceptible livestock species (108). Wild ungulate species Scimitar-horned Oryx were seropositive for FMDV in Abu Dhabi. Three outbreaks were reported and there was no mortality among 4,000 morbid animals (109). The UAE is a trade hub which makes it more susceptible to FMD.

### 3.16. Turkey

FMD is endemic in Turkey and from time to time outbreaks occur in the region (110). Serotypes O, A, and Asia 1 of FMD are reported from Turkey. Thrace district of Marmara has been declared an FMD-free region. It is the region of Turkey that borders with European Union. Disease incidents were reported to increase significantly from 2006 to 2013 (110). Between 2006 and 2013, the average annual outbreaks of FMD were 1,046 (111). The disease is reported in 30% of animals in the East and South-East Anatolia region. In 2009, seroprevalence of bovine and ovine for FMDV was 8.81% in Turkey (112). In 2011, it increased to 21.9% (113). According to economic survey reports, production losses due to FMD are significantly high (tens of millions of Turkish Lira) (114). Due to illegal animal movements and low vaccination rates, there is a high prevalence of disease in the East and Southeastern parts of Turkey. Different strategies like mass vaccination and quarantine are applied recently to overcome the disease in endemic as well as other regions of Turkey (113). By enhancing clinical surveillance programs in bordering provinces, vaccine efficacy, and control of animal movement, the Turkish government hopes to achieve an OIE status of FMD-free with vaccination by 2023 (115). Indeed, increasing border security is a crucial tactic because several studies have shown that both legal and illegal animal movement contributes to the spread of FMD.

**Figure 3:**
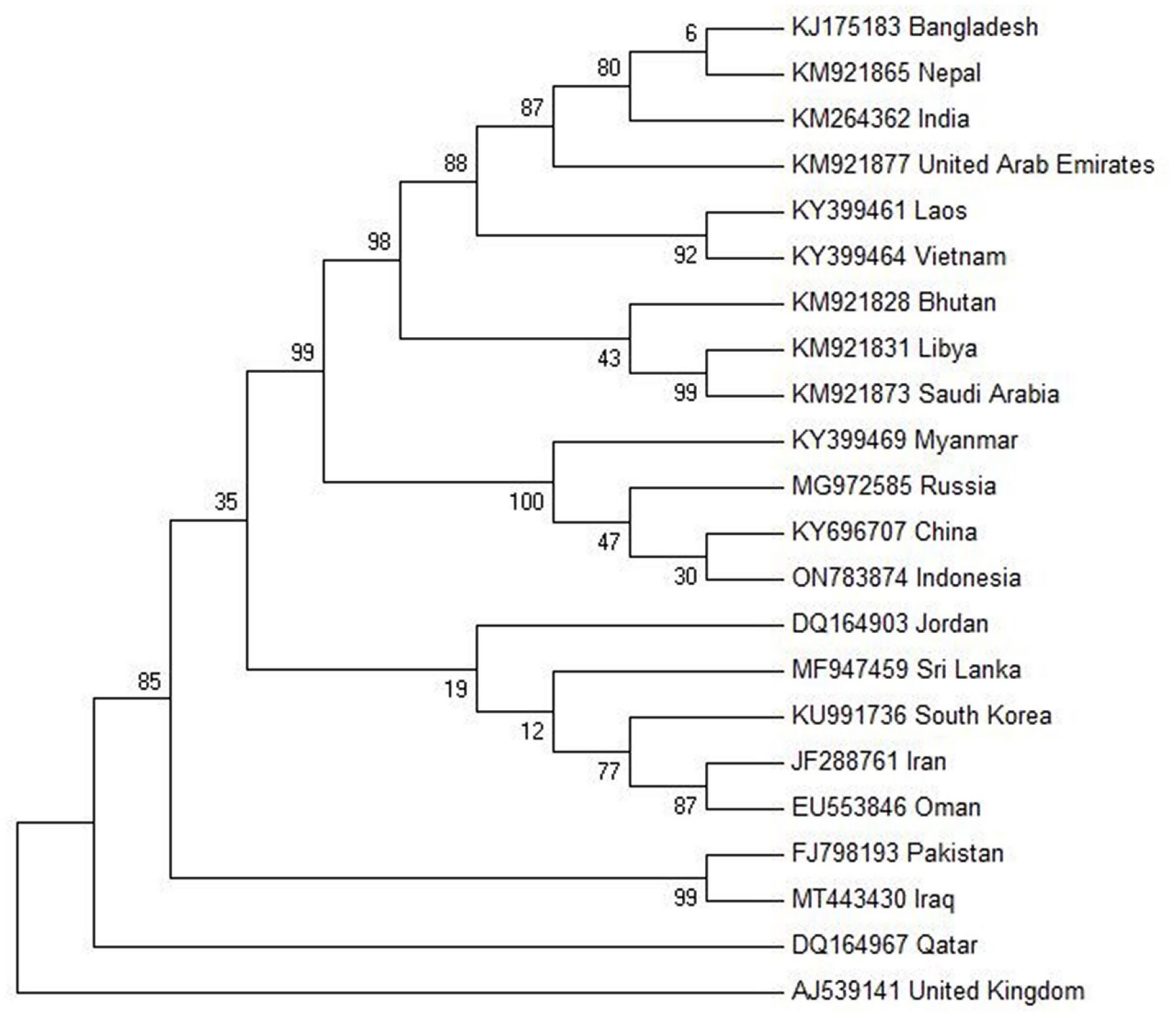
Molecular phylogenetic analysis of FMD type O VP1 protein gene by Maximum Likelihood method. Created by biorender.com.

The molecular phylogenetic analysis of FMD type O (VP1 protein) is shown in Figure 3. The evolutionary history was inferred by using the Maximum Likelihood method based on the Tamura-Nei model (116). The tree with the highest log likelihood (−3085.6853) is shown. The percentage of trees in which the associated taxa clustered together is shown next to the branches. Initial tree(s) for the heuristic search were obtained automatically by applying the Neighbor-Join and BioNJ algorithms to a matrix of pairwise distances estimated using the Maximum Composite Likelihood (MCL) approach and then selecting the topology with superior log likelihood value. The analysis involved 22 nucleotide sequences. Codon positions included were 1st + 2nd + 3rd + Noncoding. All positions containing gaps and missing data were eliminated. There was a total of 630 positions in the final dataset. Evolutionary analyses were conducted in MEGA6 (117) (Table 1).

**Table 1:** Summary of the different studies that reported the serotype of FMDV along with their prevalence percentage in particular regions.

Country name	Serotype of FMDV	References
Pakistan	Study 1: Serotype A = 4.7%, O = 70%, and Asia1 = 25%	(118)
	Study 2: Serotype A = 13.89%, O = 45.83%, Asia1 = 29.17%, AO = 9.72%, and Asia1+ O = 1.39	(30)
	Study 3: Serotype A = 24.14%, O = 65.52%, and Asia1 = 10.35%	(19)
	Serotype A = 65.25%, O = 56.25%, and Asia1 = 20.70%	
	Serotype A = 10.35%, O = 37.50%, and Asia1 = 4.60%	
	Study 4: Serotype A = 6.60%, O = 20.70%, and Asia1 = 4.60%	(119)
	Serotype A = 2.90%, O = 20.40%, and Asia1 = 4.70%	
	Serotype A = 31.60%, O = 22.40%, and Asia1 = 4.00%	
	Study 5: Serotype A = 21.91%, O = 6.84%, and Asia1 = 71.23%	(120)
India	Study 1: Serotype A = 14%, O = 67%, C = 4%, and Asia1 = 15%	(121)
	Study 2: Serotype A = 8%, O = 80%, and Asia1 = 12%	(32)
	Study 3: Serotype A = 3%, O = 92%, and Asia1 = 5%	(37)
	Study 4: Serotype A = 12.27%, O = 64.04%, and Asia1 = 19.87%	(36)
	Serotype A = 9.01%, O = 82.59%, and Asia1 = 8.40%	
Egypt	Study 1: Serotype A = 12.28%, O = 80.70%, and SAT2 = 7%	(122)
	Serotype A = 36.70%, O = 56.96%, and SAT2 = 6.32%	
	Study 2: Serotype A = 68.18%, O = 93.82%, and SAT2 = 35.23%	(82)
Iraq	Study 1: Serotype A = 57.60%, and O = 30.70%	(123)
	Study 2: Serotype A = 12.39%, O = 6.95%, SAT1 = 3.69, and Asia1 = 17.39%	(123)

Available studies have been illustrated comprehensively.

## 4. Transmission of FMDV

**Figure 4:**
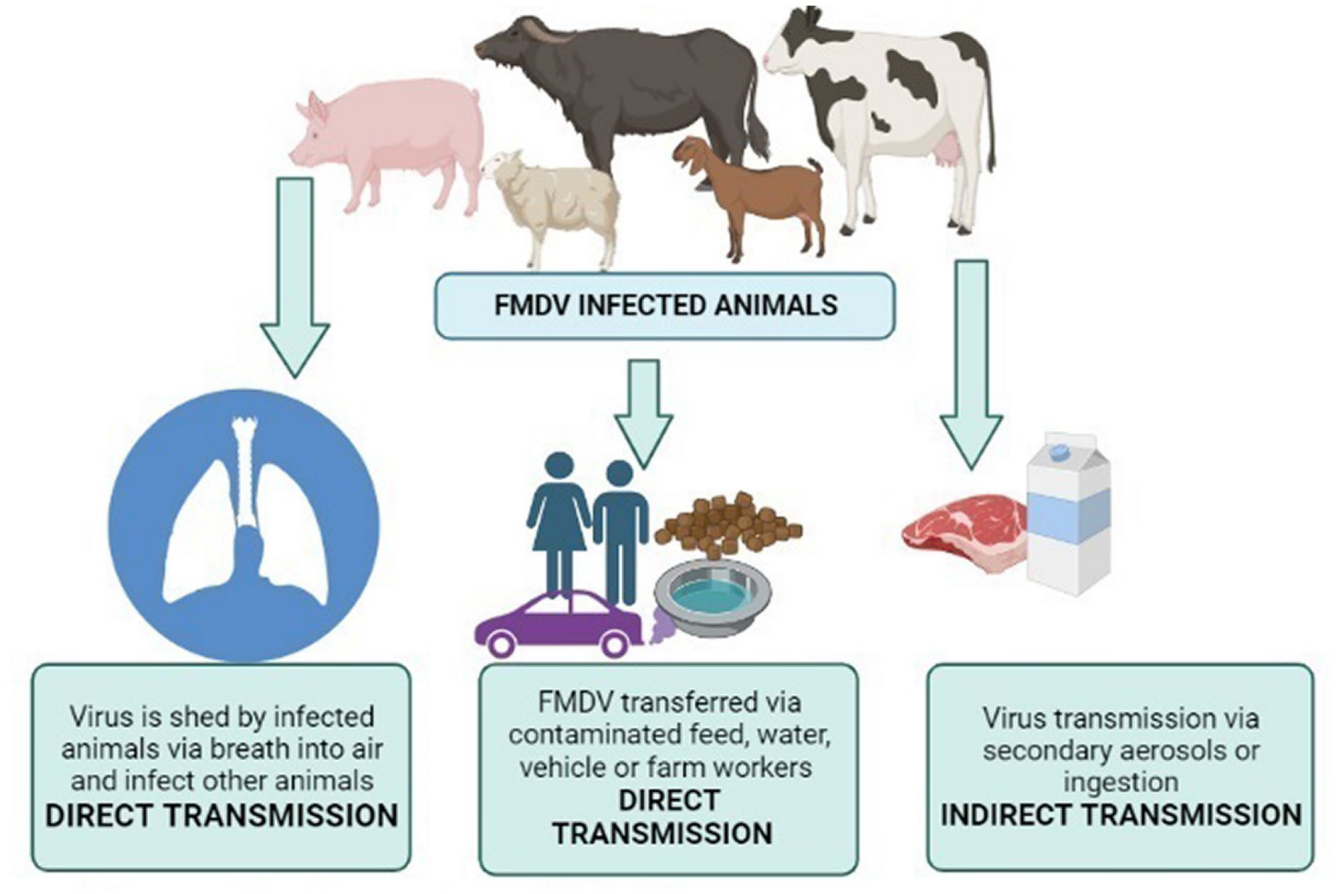
Transmission routes of FMDV. Created by biorender.com.

FMDV transmission is mainly attributed to exposure to the bodily secretions and excretions of acutely infected animals; it may be milk semen or breath (124). Susceptible animals can get the disease even with a very low dose of inhaled FMDV. This may be directly inhaled from the exhaled breath of an infected animal, or it may be the resuspension of aerosols from FMDV-contaminated materials. In comparison to other ruminants, pigs are relatively resistant to FMDV transmission via the inhalational route (124). A higher dose of viruses is required in cases other than the inhalational route, i.e., ingestion, penetration through abrasions, etc. FMDV can survive in the environment and animal products (milk and meat) for days or months. Transmission routes are illustrated in Figure 4 (produced by biorender). This ability is highly dependent on conditions like temperature and humidity in the external environment and pH for animal products (125). As soon as FMDV enters the body of an animal, a rapid immune response starts to clear the virus.

**Figure 5:**
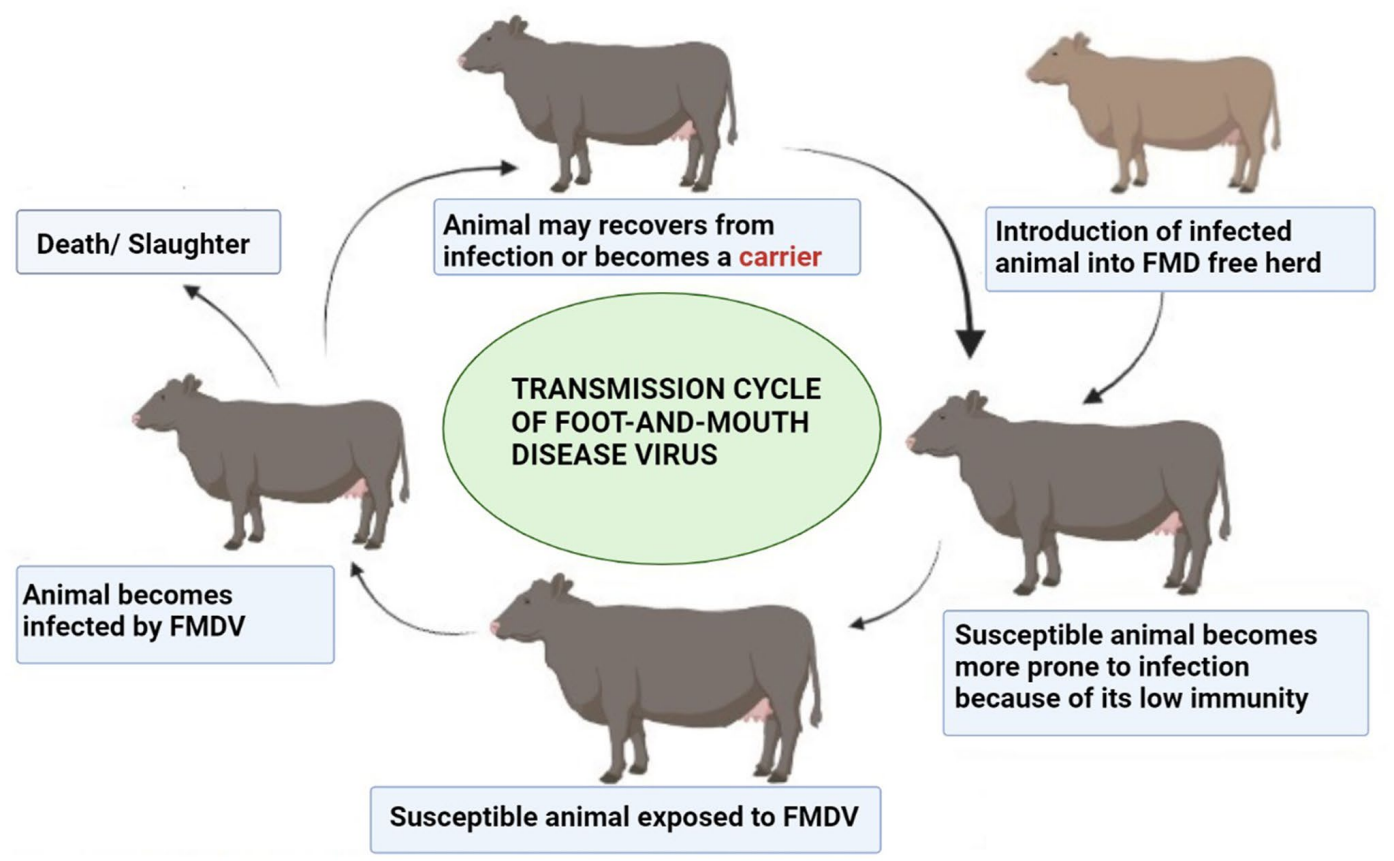
Transmission cycle of FMDV. Created by biorender.com.

Some hosts become carriers with a low level of FMDV in nasopharyngeal epithelial sites (126) and lymphoid tissue (127). FMDV can be transferred from endemic regions to disease-free zones; this explains why FMD is one of the most infectious diseases. A classic example of this wind-borne spread was seen when FMD was transferred from a pig farm in the Isle of Wight in the south of England to the North French Coast (128). Direct contact with imported animals of FMD endemic regions and traded animal products are sources of disease within FMD-free zone, and that is why trade restrictions are implemented. Carrier animals are also a source of disease persistence, and the mechanism involved in the persistence of FMDV in animals is still unclear and causing hindrances in the development of countries where the disease is endemic (129). The carrier state of animals has remained a hot topic in experimental and field studies. In a research study, a carrier state was studied in Indian cattle, and FMDV was persistent for 13 months (130). In experimentally infected cattle, cellular determinants of the carrier state were studied in host tissue samples using transcriptome analysis. The tissue was processed by laser capture microdissection (126). This indicated that persistent FMDV leads to the down-regulation of antiviral host factors. A study was conducted in Cameroon, an FMD-endemic region. Cattle herds were investigated to determine the carriers. Researchers found that the carrier state of animals decreases significantly with time and young animals are more likely to become carriers than adults (131). The transmission cycle of FMDV in a herd is illustrated in Figure 5 (produced by biorender).

## 5. Pathogenesis

To cause the disease, FMDV binds to the specified cell surface receptors and gets entry into the host cell. The receptors known to give access to FMDV in the cell are integrin (132) and heparan sulfate (HS) receptors (133), and a third receptor that has not been identified yet (134). FMDV recognizes these three receptors on the cell surface and binds to them. Receptor-mediated endocytosis occurs and the virus gains access to the host cell. Cell tropism and host range are determined by the specificity of interaction between the virus and host. The invasive efficiency of the virus is decisive in the receptor pathway used by FMDV (135, 136). Mutations can occur in the receptor binding site due to alterations in its amino acid sequence. This eventually leads to changes in the invasive manner and infection-causing ability of the virus. Serotype O of FMDV usually uses an integrin pathway for entry into the host cell (137, 138). During the experiment in cell culture, HS receptors are also utilized along with unidentified receptors to get access into the cell (139, 140). Serotype O is reported to infect the MCF10A cell line by binding to the HS receptor (141). The Spread of FMDV can be controlled once its invasion mechanisms are revealed. Integrin is a heterodimeric glycoprotein that has three domains: extracellular, cytoplasmic, and transmembrane domain. RGD is a tripeptide motif located on the VP1 of FMDV (142). For the initiation of viral infection, FMDV VP1 interacts with integrin through the RGD motif (143). Among 24 integrin receptors, αvβ3 and αvβ6 are the main receptors of FMDV (143, 144).

**Figure 6:**
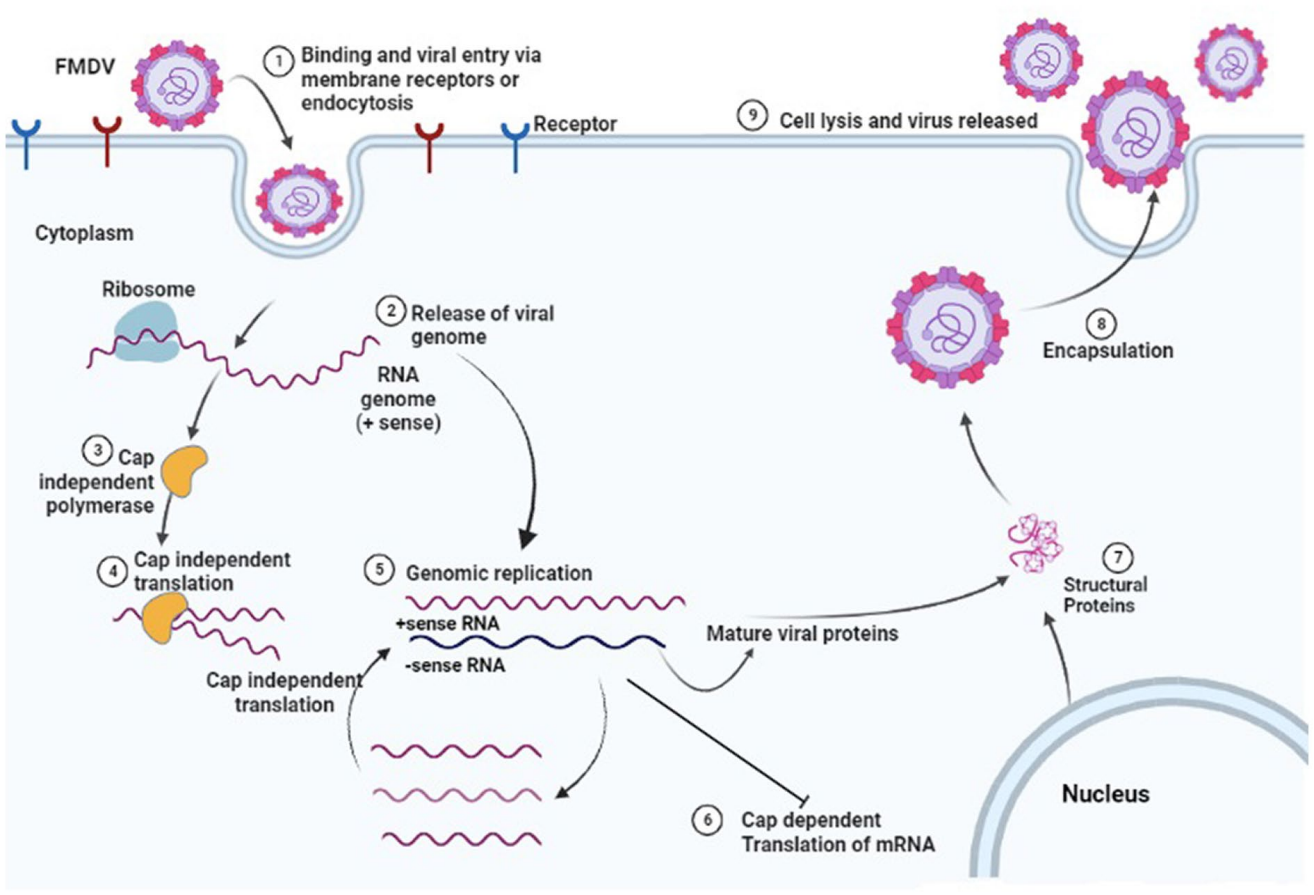
Replication cycle of FMDV in host cell. Created by biorender.com.

The binding of the VP1 protein to the integrin receptor initiated interactions among intercellular regions to intercellular junction proteins which ultimately starts the internalization process by the cell (145). Clathrin-coated pits (CCP) are formed by clathrins present that later dissociate from the cell membrane and are converted into the clathrin-coated vesicle. This vesicle takes the virion into the endosome (146) after the internalization of FMDV due to an acidic environment within which the endosome uncoating of the virion occurs. Viral RNAs are dispersed in the cytoplasm by an undiscovered mechanism (147, 148). HS is also located on the cellular surface and is a mucopolysaccharide in nature (149). VP3 interacts with O-sulfate or N-sulfate and FMDV enters the host cell via endocytosis (150). However, integrin-mediated endocytosis is faster than that mediated by HS and there is also a difference in the involved mechanism. Upon entry with the HS receptor, FMDV falls into caveola and enters the cytoplasm then ultimately goes to the recycling endosome releasing the viral RNA (141). In addition to utilizing HS and integrin receptors, there is a third type of receptor that still needs to be investigated. Researchers have claimed that certain other pathways are utilized by FMDV other than HS and integrin-mediated pathways (151, 152). Mass spectrophotometry combined with immunoprecipitation assay can be utilized to identify the third group of FMDV receptors (153). The entire replication cycle of FMDV occurs in the cytoplasm. The genetic material of a virus contains all the required information to take over the host cellular machinery and stop the synthesis of macromolecules required by the host cell. Instead of making the required macromolecules, the host machinery starts translating viral products. VP-primed RNA replication occurs with the help of RNA-dependent RNA polymerase 3Dpol (154). It forms complementary negative-strand RNA molecules by transcribing the positive-strand RNA. Multiple positive strands of RNA are generated by 3Dpol which either enters the central dogma of translation and replication of RNA or forms a new virus by getting packaged by capsid proteins. Continuous replication of the virion leads to cell lysis and they are finally released to infect the neighboring cells (154). The whole mechanism of the pathogenesis of FMDV is illustrated in Figure 6 (produced by biorender).

## 6. Pathophysiology of the disease

The disease is clinically characterized in cattle by high-grade fever (~40°C) leading to vesicular lesions on the hard palate, lips, gums, tongue, dental pad, muzzle, interdigital cleft, coronary band, and teats. Acutely infected animals prefer to lie down, stamp on their feet, and salivate profusely. Erosions are formed in the mouth upon the coalescence of ruptured oral vesicles and heal approximately in 11 days (155). Healing duration is longer in vesicles present on feet and are more prone to bacterial infection, leading to chronic lameness. Teat lesions may give rise to mastitis due to secondary bacterial infection. After the development of vesicular lesions, the animals quickly lose condition and milk production capacity which can persist for a longer duration (156). Sometimes, young calves die without showing clinical signs, and virus-induced myocardial damage is observed upon necropsy. In pigs, fever is usually up to 41.5°C with blanching around the coronary band and mild lameness. Infected pigs become anorexic, lethargic, and huddle among other pigs. Vesicles develop on the heels of the foot, snout, mandible, tongue, and coronary band. Pigs that are housed on rough surfaces may develop vesicles on knees ad hocks (156). In recovered pigs, lameness is seen due to the complete sloughing of the horn of the foot. Myocarditis is more frequent in pigs than in calves. Young pigs occasionally die without showing any clinical signs due to myocarditis (157).

FMD is usually inapparent in sheep and goats in terms of the appearance of clinical signs. However, the first observed clinical sign in the case of sheep and goats is lameness followed by fever and vesicle formation (158). Vesicles appear on heel bulbs, interdigital cleft, coronary band, and mouth. In lactating animals, vesicles may appear on teats and are rarely observed on the vulva and prepuce. Infection may make the animals prone to other viral diseases such as sheep pox, goat pox, and peste des petits ruminants (159). Sudden death in young ones due to myocarditis is also observed in sheep and goats. Experimentally infected camels are reported to have mild clinical signs, but they tend to get severe infections resulting in mouth lesions, excessive salivation, and sloughing of the footpad and skin of the tarsal and carpal joints. Water buffalo get lesions on the mouth and foot, but they are less severe and heal faster than cattle (157). FMD clinical signs in wildlife resemble the signs in their domestic counterpart. The sloughing of the antlers toe and horn is reported due to FMD. For epidemiological investigations of FMD, the aging of lesions can play an important role. Brochures are available for practitioners to estimate the age of clinical lesions of FMD.

### 6.1. Lab investigations in FMD patients

Certain changes occur in the blood profile of FMD-infected patients. The serum of infected animals was analyzed. Interleukin-1 (IL-1), tumor necrosis factor-alpha, interferon-gamma (IFN-γ), interleukin-6, serum amyloid A protein, lactoferrin, mannose-binding lectin, and monocytes chemo-attractant protein-1 were elevated significantly while interleukin-4 and interleukin-10 concentration was reduced in affected cows (160). Another study suggested that interferon levels and MHC levels both significantly drop during FMDV infection, which eventually aids in the progression of the disease. Interleukin-2, IL-12, IL-15, and IL-18 reduces in number while IL-10 increases (153).

## 7. Treatment and prevention

FMD is an economically significant and highly contiguous transboundary disease. For its control and prevention, it is necessary to have some reliable diagnostic tools and proper treatment. The treatment of FMD has still not been discovered (161). The absence of specific treatments gives rise to the application of supportive treatment. Antibiotics along with flunixin meglumine and mild disinfectants are used as conventional methods of treatment. Ethnoveterinary practices have been widely documented for the treatment of FMD (161). Natural soda ash solution, finger millet flour, and honey have been used for the washing of lesions (162). The use of interferons is employed to prevent the disease in swine (163) and cattle (164). A combination of FMD vaccine along with interferon is also used to protect the animal from disease (165). 2-C-methylcytidine (166) and ribavirin (167) are the antivirals used as a prophylaxis measure to prevent the disease in susceptible animals. In endemic regions where slaughtering the infected animal is not possible, dressing of lesions is done and antibiotics are given to prevent secondary bacterial infection. Tetracycline is used via the parenteral route as it is a broad-spectrum antibiotic that can prevent bacterial infection (168). In zones where FMD is endemic, a repeated vaccination strategy is adapted to eliminate the disease. Livestock is kept separate from wildlife and animal movement is controlled to prevent the disease chances (169). However, it is not feasible to adapt the test and slaughter policy for the control of FMD in endemic regions due to economic and social problems (170).

## 8. Future perspective

Infectious diseases are critical health problems in both animals and humans, which cause economic losses and severe illnesses (171–175). FMD is a hot topic nowadays due to its disastrous results in the livestock sector, and its control is necessary to ensure the safety of livestock as well as the economy of the country. In the future, due to rapid genetic variation among serotypes, the virus will evolve more, and it will cause several outbreaks in the endemic regions. Measures should be taken depending on the status of the disease whether it is endemic or not. A trained veterinary staff in disease control, good infrastructure, better governance, diagnostic testing with high sensitivity and specificity, and well-stocked laboratories should be developed to control the disease. A good monitoring and surveillance system must be there to prevent the outbreak at a bigger level. FMDV does not offer cross-protection which is why strain-specific mass vaccination must be adopted in endemic regions to prevent the disease and its future outbreaks. A consortium should be developed between virologists, pathologists, and surveillance reporters, and reports should be developed to create effective vaccines and preventive measures; otherwise, the disease will remain endemic and will cost billions of dollars, which ultimately becomes a hindrance to the development of a country. Furthermore, food security issues will rise with decreasing production of animal products. *Per capita*, the availability of milk and meat to individuals will decrease and ultimately lead to malnourishment. FMD has zoonotic implications which will further deteriorate the situation so it should not be overlooked. It is advised that dairy farmers, laboratory workers, animal handlers, veterinarians, and persons in contact with wild ungulates (zoo workers) take precautionary measures to prevent the disease.

## 9. Conclusion

FMD is among the endemic diseases of livestock in Asian countries. It has seven serotypes, and serotype O is the most prevalent among those countries. FMDV outbreaks have been occurring for many decades in South Asian countries (Pakistan, India, Bangladesh, and Afghanistan), China, Russia, Kazakhstan, Mongolia, Egypt, Iran, Iraq, Sudan, Oman, Kuwait, Saudi Arabia, and Turkey. FMDV has direct and indirect transmission, and carrier animals help in the persistence of the virus. The pathogenic cycle of the virus starting upon entry into the host leads to FMD. Ruminants infected with FMD show vesicles on the foot and mouth and a high mortality rate is noted in young animals with tiger heart signs (myocarditis). No specific treatment is available for FMD, but symptomatic treatment is done in infected animals. The disease is of economic concern as trade restrictions are implemented which ultimately harms the country’s earnings via export, so preventive strategies must be adapted. The recommendation is to improve the disease surveillance system along with disease reporting, detection, and quick response to tackle the outbreak of foot-and-mouth disease in animals.

## Author contributions

All authors listed have made a substantial, direct, and intellectual contribution to the work and approved it for publication.

## Conflict of interest

The authors declare that the research was conducted in the absence of any commercial or financial relationships that could be construed as a potential conflict of interest.

## Publisher’s note

All claims expressed in this article are solely those of the authors and do not necessarily represent those of their affiliated organizations, or those of the publisher, the editors and the reviewers. Any product that may be evaluated in this article, or claim that may be made by its manufacturer, is not guaranteed or endorsed by the publisher.
